# A Review of Current Management of Vitreomacular Traction and Macular Hole

**DOI:** 10.1155/2015/809640

**Published:** 2015-03-03

**Authors:** Alfredo García-Layana, José García-Arumí, José M. Ruiz-Moreno, Lluís Arias-Barquet, Francisco Cabrera-López, Marta S. Figueroa

**Affiliations:** ^1^Clínica Universidad de Navarra, Avenida de Pío XII 36, 31008 Pamplona, Spain; ^2^Hospital Vall d'Hebron, Passeig de la Vall d'Hebron, 119-129, 08035 Barcelona, Spain; ^3^Hospital Universitario de Albacete, Avenida de Almansa, s/n, 02006 Albacete, Spain; ^4^Hospital de Bellvitge, C/Feixa Llarga, s/n, L'Hospitalet de Llobregat, 08907 Barcelona, Spain; ^5^Complejo Hospitalario Universitario Insular Materno Infantil de Gran Canaria, Avenida Marítima del Sur, s/n, 35016 Las Palmas de Gran Canaria, Spain; ^6^Hospital Universitario Ramon y Cajal Carretera de Colmenar km 9, 28034 Madrid, Spain; ^7^Vissum Madrid, Santa Hortensia 58, 28002 Madrid, Spain

## Abstract

The paper presents a review of the sequence of events of posterior vitreous detachment (PVD), vitreomacular adhesion (VMA), vitreomacular traction (VMT), and macular hole (MH) from their pathophysiological aspects, clinical features, diagnostic implications, and current management strategies. A treatment algorithm to be used in clinical practice in patients with VMA, VMT, and MH based on the presence of symptoms, visual acuity, associated epiretinal membrane, and width of the vitreous attachment is presented. Observation, pharmacologic vitreolysis with ocriplasmin, and surgical treatment are positioned as treatment options in the different steps of the therapeutic algorithm, with clear indications of the paths to be followed according to the initial presenting manifestations and the patient's clinical course.

## 1. Introduction

Posterior vitreous detachment (PVD) is a common phenomenon frequently related with aging of ocular structures [1]. The presence of persistent vitreomacular adhesions exerting tractional forces (vitreomacular traction, VMT) may be associated with the development of macular hole (MH) [2, 3]. These alterations in the symptomatic phase may cause visual disturbances, including photopsia, metamorphopsia, blurred vision, and decreased visual acuity, which in addition of causing visual-related problems may affect negatively the patient's health-related quality of life [4]. The introduction of optical coherence tomography (OCT) has allowed a more accurate visualization of the macular anatomy and better knowledge of the pathophysiology of the process, including measurement and assessment of MH characteristics [5–7], facilitating treatment decision-making.

### 1.1. Anatomy of the Vitreous and the Vitreoretinal Interface

The vitreous gel is responsible for the stabilization of the eyeball through collagen fibers (mainly type II collagen). Collagen fibers are running in an anteroposterior direction through the vitreous center, convering in the anterior vitreous base, and inserting into the posterior vitreous cortex [8]. Spaces between the collagen fibrils are maintained by the protein opticin and the glycosaminoglycan chondroitin sulphate [4]. Spaces between the collagen fibrils are mostly filled with water (98% of the vitreous gel component) and hyaluronic acid, which provides the gel-like consistency of the vitreous.

The vitreoretinal interface is a complex anatomical structure composed by the union between the retina and the vitreous [9]. Densely packed collagen fibrils of the posterior vitreous cortex (100–300 *μ*m in thickness) lie over the macula and are superficially inserted into the internal limiting membrane (ILM) of the retina by means of adhesion molecules, such as laminin, fibronectin, and proteoglycans, which interact with opticin in the vitreous gel [4]. Adherences are more firmly attached to the retina at the vitreous base, optic disc, and fovea, as well as along the major retinal blood vessels. The vitreomacular junction has an annular shape, with a diameter of 3-4 mm.

The set of events that occur as the eye ages are associated with a series of physiological changes in the vitreous gel, with progressive liquefaction (at the age of 80, around 50% of the vitreous gel has been liquefied) and gradual destruction of the collagen-hyaluronic acid network [10]. This occurs as a result of the development of fluid-filled pockets beginning in front of the macula, which over the time coalesce and enlarge, resulting in a weakened adhesion between the vitreous and the retina. This gradually predisposes to PVD, defined as separation of the posterior cortex from the ILM of the retina, which represents the final step of the normal vitreous aging process [11, 12].

PVD is an insidious process that occurs over the course of months or years, being asymptomatic in many cases until complete separation of the vitreous from the macula and optic nerve, which is the final stage. However, the anterior attachment to the vitreous base is very strong and remains for a long time. Acute symptoms of complete PVD include photopsia (by vitreous traction on the peripheral retina) and floaters by condensation of the vitreous collagen, glial tissue, or blood around the optic nerve [4].

Studies in healthy adults have shown that focal perifoveal PVD occurs in 50% of subjects aged between 30 and 39, whereas complete PVD is found in 50% of subjects aged 70 years or older [13, 14]. In addition to advanced age, PVD is more frequent in postmenopausal women by the effects of decreased estrogens on the connective tissue (within the vitreous gel), as well as in the presence of myopia [4].

The normal process of PVD due to vitreous aging may be complicated by the presence of vitreomacular adhesions between the cortex and the macular area, resulting from vitreous syneresis [15]. These adherences may be focal or extensive, affecting the foveola only or a wide region of the macular area and the optic disc. Simple vitreomacular adhesion (VMA) is not associated with distortion of the macular architecture. However, these adherences may exert traction forces on the macula (VMT), increasing secondarily during ocular saccades [16]. This may cause retinal distortion and foveal detachment. On the other hand, continuous anteroposterior traction by vitreous contraction may cause alterations, such as cystoid macular edema.

Full-thickness MH is an anatomic defect in the fovea with interruption of all neural retinal layers [17]. With the use of high-resolution OCT, it has been shown that idiopathic MHs are initiated during perifoveal PVD as a consequence of the dynamic anteroposterior VMT process. This anteroposterior VMT may cause intraretinal cavitation with progression to dehiscence of the outer retinal layers and complete detachment of the cyst roof giving rise to a full-thickness defect. Stages of the development of MH from focal VMT to complete aperture together with accompanying symptoms have been described by Gass [18, 19].

The introduction of enzymatic vitreolysis [20], which can result in the liberation of VMT, opens highly interesting new perspectives in this field.

### 1.2. Risk Factors and Epidemiology of VMT

A few studies have been specifically addressed to the epidemiology of idiopathic VMT due to the overlapping of this condition with other ophthalmological diseases [4]. A prevalence of isolated idiopathic VMT, without MH, has been estimated as approximately 22.5 cases per 100 000 of the general population, with an incidence of 0.6/100 000 persons-year [21]. In different observational and intervention studies, the mean age of patients with VMT was around 65–70 years (range 48–64), with a predominance of females [4, 15].

Regarding the prevalence of MH, it has been reported around 0.1 to 0.8 in adults aged >40 years [22], with an age-adjusted incidence of 7.8 cases per 100 000 of the general population per year [23]. Also, the risk of development of MH in the fellow eyes, without manifestations of PVD, has been estimated at around 7–12% after 5 years and 17% at 20 years [4].

Approximately two-thirds of patients with MH are women, and the disease is unilateral in 80% of cases. An increase in serum fibrinogen level has been reported as a risk factor for MH [24], whereas the use of estrogen replacement therapy in women decreases the risk [4]. In subjects with myopia, the prevalence of MH may reach 6% [25].

## 2. Diagnosis, Definition, and Classification of VMT and MH

Now, nearly two decades since the introduction of OCT, it is possible to assess and define the pathologic progression of disorders affecting vitreoretinal interface with a high level of accuracy and reproducibility. On the basis of OCT-derived anatomic findings, a unified classification scheme for disease of the vitreomacular interface has been developed.

With this purpose, a group of experts in diseases of the vitreoretinal interface (*International Vitreomacular Traction Study Group,* IVTS) [26] have proposed a classification system for diseases of the vitreomacular interface. This evidence-based classification is a clinically applicable system that is predictive of therapeutic outcomes and is useful for the execution and comparative analysis of clinical studies.

### 2.1. VMA

VMA represents a specific stage of partial vitreous detachment in the perifoveal area without retinal abnormalities. In previous classifications, VMA is the equivalent of a stage 1 PVD [11, 15, 27, 28]. VMA is characterized by elevation of the cortical vitreous above the retinal surface, with the vitreous remaining attached within a 3 mm radius of the fovea (as defined arbitrarily). The angle between the vitreous and the inner retinal surface is acute, and the retina displays no abnormalities in contour or morphological features of OCT. VMA is not accompanied by visual impairment and may be considered a normal finding in the natural course of PVD. Also, VMA may be subclassified by the size of the adhesion into focal (≤1500 *μ*m) or broad (>1500 *μ*m). The cutoff of 1500 *μ*m corresponds to the area of increased vitreous adhesion to the fovea. VMA usually resolves spontaneously as part of the normal process of PVD, although it may progress to VMT and, for this reason, periodic monitoring with OCT is necessary.

### 2.2. VMT

Macular traction due to progression of PVD causes anatomic changes in contour of the foveal surface, intraretinal pseudocyst formation, and disappearance of foveolar depression, which typically results in reduced or distorted vision. The following anatomic criteria [26] should be present at least in one OCT image to classify an eye as having VMT: (a) evidence of perifoveal vitreous cortex detachment from the retinal surface, (b) attachment of the vitreous cortex to the macula within a 3 mm radius of the fovea, and (c) association of this attachment with distortion of the foveal surface, intraretinal structural changes, foveal detachment from the retinal pigment epithelium (RPE), or a combination of these findings, without full-thickness interruption of all retinal layers. VMT can also be subclassified as focal or broad (using the same cutoff of 1500 *μ*m) depending on the width of the vitreous attachment. Distortion of the foveal profile, formation of intraretinal cysts, intraretinal cavitation, subretinal fluid, and, even, RPE detachment can be observed.

On the other hand, proliferation of residual of vitreous tissue provides the anatomic substrate to form an epiretinal membrane (ERM), which in turn may appear at any stage of vitreous separation. ERM is composed of retinal pigment epithelial cells, fibroblasts, and macrophages. ERM may be associated with peripapillary vitreoretinal traction with blurred disc border.

Although spontaneous resolution of VMT may occur, traction on a large surface or the presence of ERM is poor prognostic factor. In symptomatic patients, enzymatic vitreolysis or vitrectomy may be indicated.

### 2.3. MH

As stated above, full-thickness MH is an anatomic defect in the fovea featuring interruption of all neural retinal layers. The observation of the anatomic opening on several scans through the fovea is an unequivocal sign. According to the aperture size, MHs are considered small (<250 *μ*m), medium (250 to 400 *μ*m), and large (diameter > 400 *μ*m). Nearly half of full-thickness MHs are large at the time of diagnosis [26]. Also, on the basis of OCT findings, MH can be categorized according to the presence or absence of VMT. Only patients with MH and concomitant VMT are candidates for pharmacologic vitreolysis. The correlations between MH stages commonly used in clinical practice and OCT-based images proposed by the IVTS group are shown in Table 1.

Moreover, MH can be subdivided into idiopathic and secondary. Primary MH results from vitreous traction on the fovea from anomalous PVD (incomplete or inadequate separation of the vitreoretinal interface), whereas secondary MHs are caused by other pathologic conditions and do not have preexisting or concurrent VMT. Secondary MHs have been reported in cases of blunt ocular trauma [29], lightning strike [30], high myopia [25, 31], macular schisis [32], macular telangiectasia type 2 [33], occlusion of the central retinal vein, diabetic macular edema, uveitis, and age-related macular degeneration [26].

## 3. Treatment Options

### 3.1. Observation

The availability of OCT, particularly spectral domain OCT (SD-OCT), has allowed a more accurate diagnosis and precise assessment of adhesion of the vitreous to the macula, differentiating VMA from VMT [26]. Before the introduction of OCT, only patients with advanced VMA could have been diagnosed by biomicroscopy and, for this reason, the rates of spontaneous deterioration reported were high (64%) [34].

**Figure 1:**
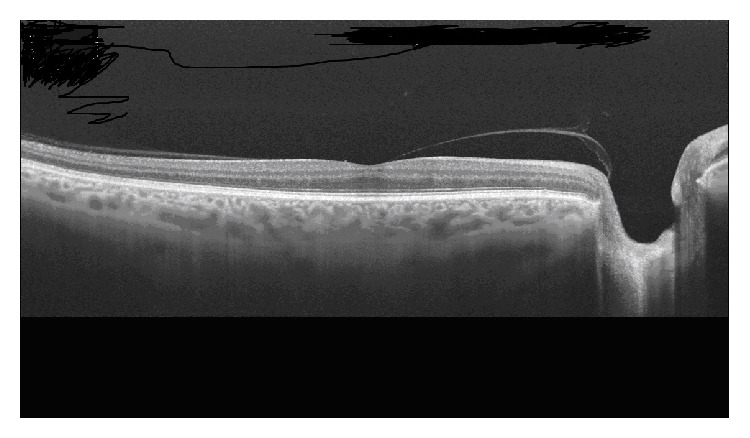
Horizontal image with swept-source OCT at the foveal level showing posterior vitreous detachment, which remains adhered to the fovea and the papillary edge.

Studies using SD-OCT have shown that incomplete vitreous detachment with persistent vitreoretinal adhesions is more frequently observed than by clinical diagnosis. During the physiological process of PVD, the vitreous remains attached to the foveal region in the last stages (Figure 1), so that, VMA can be considered a normal stage in the natural history of PVD associated with vitreous aging [13, 26]. Only when symptoms are present or when foveal anatomic changes are observed, VMA can be considered a pathological process [34].

Recently, John et al. [35] investigated the spontaneous clinical course in 106 eyes of 81 patients identified as having VMA by SD-OCT and classified into three grades, with a mean follow-up of 18 months (range 1 to 91). The authors defined three grades to classify adherence: Grade 1 (41%) was incomplete cortical vitreous separation with attachment at the fovea, Grade 2 (52%) was the Grade 1 findings and any intraretinal cysts, and Grade 3 (7%) was the Grade 2 findings and the presence of subretinal fluid. By the last follow-up, spontaneous release of VMA occurred in 32% of cases (34 eyes, in 30%, 30%, and 57% of Grades 1, 2, and 3, resp.). No changes were observed in 23, 31, and 2 eyes (52% of the total), and progression occurred in 7, 8, and 1 eye of Grades 1, 2, and 3, respectively (16% of the total). The authors conclude that the clinical course of patients with VMA managed by initial observation was generally favourable in asymptomatic patients or with minimal symptoms of VMT.

**Figure 2:**
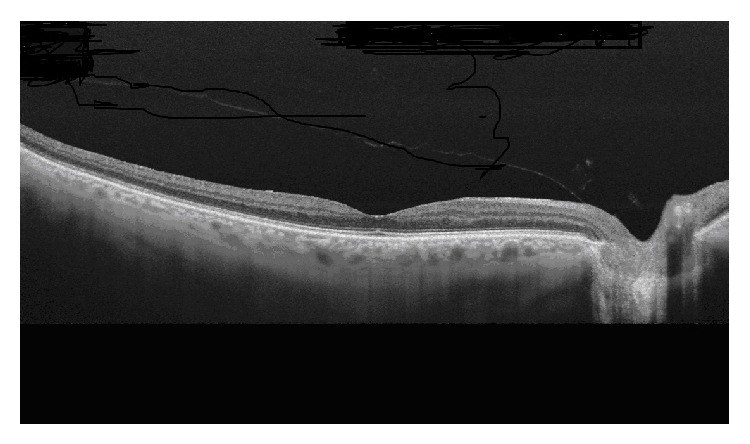
Study of the retinal surface with swept-source OCT showing posterior vitreous detachment, which remains attached at the papillary level.

Studying the retinal surface with SD-OCT, it has been observed that PVD appears to begin in the perifoveal region, with a slow clinical course taking even years until complete separation of the vitreous from the papilla (Figure 2). In most patients this process is asymptomatic but, in some cases, PVD may be complicated by macular pathology [2]. In a OCT study of eyes with macular edema secondary to VMT, published in 2012, complete and spontaneous resolution of traction was observed in 53% of eyes [36].

The clinical course of VMA, particularly in asymptomatic patients, remains to be fully elucidated. Systematic examination with SD-OCT has been associated with an increase in diagnostic rates and has allowed assessing more accurately the course of this physiological process that may evolve into VMT, remain stable, or resolve spontaneously. Therefore, in the presence of a VMA syndrome, the first approach is to reexamine the patients using OCT at a period of 3 months. Even in cases of evolution to a VMT syndrome, observation still remains an option, given the possibility of spontaneous resolution of VMT.

### 3.2. Pharmacologic Vitreolysis: Ocriplasmin

Ocriplasmin is a truncated form of human plasmin that induces liquefaction of the vitreous and separation of the vitreous cortex from the retinal surface due to proteolytic activity against main components of the vitreomacular adhesion.

#### 3.2.1. Results of Clinical Trials of Ocriplasmin and Initial Data in Clinical Practice

The efficacy and safety of ocriplasmin have been evaluated in two pivotal, phase 3 clinical trials (TG-MV-006 y TG-MV-007) carried out in the United States and Europe [20]. Both studies were very similar except for the ratio of randomized assignments to ocriplasmin and placebo, which was 2 : 1 in the TG-MV-006 study and 3 : 1 in the TG-MV-007. Overall, 652 patients were randomized, 464 were assigned to treatment with a single intravitreal injection of ocriplasmin (125 *μ*g) and 188 to a placebo intravitreal injection. The primary endpoint was the pharmacologic resolution of VMA at day 28, as determined by OCT. Secondary endpoints included the percentage of patients with complete PVD and nonsurgical closure of full-thickness MH at day 28. Eligible patients had symptomatic focal VMA as seen on OCT and a best-corrected visual acuity of 20/25 or less. Exclusion criteria were high myopia (more than −8 diopters or axial length > 26 mm), prior vitrectomy or prior laser photocoagulation of the macula, and other eye diseases that may affect visual acuity. Patients with a MH > 400 *μ*m in diameter were also excluded. Of note, the presence of an ERM was not a criterion for exclusion.

At day 28, VMA resolved in 26.5% of ocriplasmin-injected eyes and in 10.1% of placebo-injected eyes (*P* < 0.001). The between-group differences did not change substantially at 6 months (26.9% ocriplasmin versus 13.3% placebo, *P* = 0.001). Also, 72% of patients with resolution of VMA showed the release during the first seven days. Results of adhesion release were better in patients without ERM (37.4% in the ocriplasmin group versus 14.3% in the placebo group, *P* < 0.001).

With regard to secondary variables (day 28), 13.4% of patients treated with ocriplasmin showed total PVD as compared to 3.7% of those treated with placebo (*P* < 0.001). Also, nonsurgical closure of full-thickness MH was achieved in 40.6% of ocriplasmin-treated patients and in 10.6% of placebo-treated patients (*P* < 0.001).

According to the investigator's criteria, all patients could be treated with vitrectomy in the framework of the study if macular disease did not resolve. At 6 months, vitrectomy was performed in 17.7% of patients in the ocriplasmin group and in 26.6% of those in the placebo group (*P* = 0.02). At 6 months, there were statistically significant differences in favour of ocriplasmin in the gain of two or more lines (23.7% versus 11.2%, *P* < 0.001) or three or more lines (12.3% versus 6.4%, *P* = 0.02).

Important safety-related problems were not observed. Most adverse events were related to the development of PVD induced by ocriplasmin injection (floaters and photopsia). There was a slightly higher incidence of retinal tears or detachments in the placebo group, which was attributed to the higher proportion of patients treated by means of vitrectomy in this group.

The favourable results obtained in both clinical trials allowed approval of the use of intravitreal injection of ocriplasmin for the treatment of symptomatic VMT and MH by the Food and Drug Administration (FDA) in the United States, in November 2012, and by the European Medicines Agency (EMA) in May 2013.

Outside the context of clinical trials, recent reports have provided data of the use of ocriplasmin in daily practice. In a retrospective study [37], 17 patients with symptomatic VMT were treated with a single intravitreal injection of ocriplasmin 0.125 mg. By day 28, resolution of VMT was verified by SDOCT in eight patients (47.1%), 7 of which (87.5%) had already experienced release by day 7. Those who did not have traction release showed no statistically significant change in VMA diameter. Four of the five patients (80%) with MH at baseline experienced resolution of their MH after injection. Significant differences in visual acuity were not observed (20/49 at baseline and 20/46 at final follow-up). It should be noted that patients meeting the four positive predictor criteria (younger than 65 years, no ERM at baseline, traction <1500 *μ*m, and phakic lens status) showed a response rate of 75% (three of four eyes). Transient outer segment ellipsoid zone loss was documented in 7 cases (41.1%) and subretinal fluid presence following injection was noted in 5 cases (29.4%) [37].

In another study of 19 patients with symptomatic VMA treated with intravitreal ocriplasmin, resolution of VMA was observed in 8 cases (42%) [38]. Results were significantly affected by lens status, with adhesion release in 53% of phakic patients, whereas no case of resolution of adhesions was observed in pseudophakic patients. Also, closure of MHs after treatment was found in 3 of 6 patients (50%). Visual acuity remains stable, with a slight tendency towards improvement in the majority of cases. Only one patient showed an important loss of visual acuity (from 20/70 to 20/200) due to progression of VMT to a full-thickness MH. Significant adverse events were not recorded.

#### 3.2.2. Safety Profile

The safety profile of ocriplasmin has been evaluated in the two pivotal trials [20]. The proportion of patients who had any ocular adverse event was 68.4% in the ocriplasmin group and 53.5% in the placebo group (*P* < 0.001). This difference was driven primarily by adverse events known to be associated with PVD. The most common complications included vitreous floaters (ocriplasmin 16.8% versus placebo 7.5%, *P* = 0.002), photopsia (11.8% versus 2.7%, *P* < 0.001), blurred vision (8.6% versus 3.2%, *P* = 0.01), and visual impairment (5.4% versus 1.6%, *P* = 0.02). Most of these adverse events were transient and mild in severity. There were no differences between the groups in terms of severe ocular adverse events, including development of MH (5.2% versus 8.6%), retinal detachment (0% versus 1.6%), and reduced visual acuity (0.6% versus 0.5%).

However, since the real-world use of the drug began, there have been some unfavourable reports of visual disturbances after ocriplasmin injection, including transient but profound visual decline, raising concerns regarding its safety. Of 976 patients receiving ocriplasmin injection in clinical trials, 9 patients were reported to have experienced an acute decrease in vision, some to the hand motions level, within 24 hours of injection [39]. In 8 of these 9 patients, vision returned to baseline with a median recovery time of 2 weeks. In the clinical trials of ocriplasmin, dyschromatopsia, and electroretinographic (ERG) changes occurred in a significantly greater number of eyes treated with ocriplasmin than in eyes receiving placebo [20, 40].

Freund et al. [41] recently reported a single case report demonstrating changes seen in the outer photoreceptor segments by SD-OCT. The disruption occurred in the ellipsoid zone and was reversible. Since the clinical trials [20] used only time-domain OCT with inferior resolution to SD-OCT, it is possible that these cases may have been overlooked.

**Figure 3:**
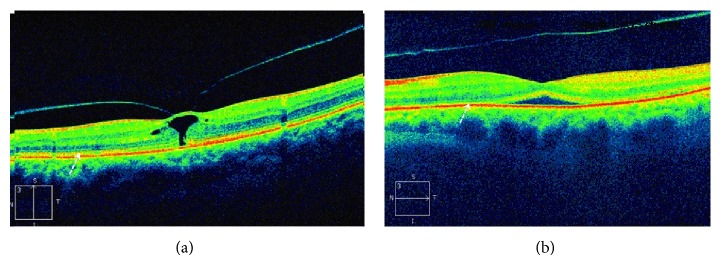
(a) Focal VMT. The arrow points to the ellipsoid zone. (b) Release of VMT after injection of ocriplasmin. A severe disruption in the ellipsoid zone is shown (by courtesy of Dr. Peter K. Kaiser, Cleveland Clinic, Cleveland, OH, USA).

In another study in which 17 patients were included [37], almost all the patients who responded to the treatment (7/8) had ellipsoid zone changes on the SD-OCT (Figure 3). These patients also had transient reduction of visual acuity and demonstrated subretinal fluid during the release process with almost the exact time course as the loss of the OS ellipsoid zone. The loss of the OS ellipsoid zone occurred after an average of 5 days after injection of ocriplasmin and the mean time of resolution on OCT was 29.3 days. The occurrence and resolution of subretinal fluid occurred at an average of 4.8 days and 30 days after injection, respectively. However, in a retrospective review of 62 eyes with symptomatic VMA treated with ocriplasmin, subretinal fluid appeared in 37% of cases, with persistence of fluid in 30% of cases after 5 months of follow-up [42]. Other studies have also shown resolution of the ellipsoid zone changes in most patients within weeks or months after ocriplasmin injection [43, 44].

Alteration of the ellipsoid zone on SD-OCT and a significant decrease in ERG amplitudes have been also reported in two patients with release of symptomatic VMT after ocriplasmin injection [45, 46]. It is possible that this transient effect of the medication may be due to a diffuse enzymatic effect of the protease on the photoreceptors or the retinal pigment epithelium throughout the retina. The greater reduction in scotopic function compared with photopic function suggests that rod photoreceptors may be more susceptible than cone photoreceptors to the effects of ocriplasmin. If this transient affect occurs for both rods and cones, it may explain the dyschromatopsia, contrast sensitivity changes, dark adaptation issues, and ERG changes reported in the ocriplasmin clinical trials.

An ongoing phase 3b, 24-month randomized clinical trial which will evaluate ERG and microperimetry in ocriplasmin-treated eyes compared to sham, will provide additional clarifications on the observed EGR changes and dyschromatopsia events (*OASIS Study*; NTC01429441) already reported.

### 3.3. Surgical Treatment

#### 3.3.1. Peeling of the Internal Limiting Membrane

Surgery of idiopathic MH with ILM peeling is a very safe procedure, with good anatomic and functional results and scarce postoperative complications [47]. Data provided by clinical trials have shown that peeling of the ILM significantly increases MH closure rates and is also associated with significantly lower percentages of reoperation and reopening. Therefore, ILM peeling is a cost-effective technique and the procedure of choice for all patients with idiopathic full-thickness MH susceptible to undergo surgical treatment [48–53].

Broad ILM peeling to the vascular arcades is recommended, so that tangential traction forces on the MH edges are removed facilitating approximation and closure [54]. In cases of large MH (>400 *μ*m) with increased risk of failure of primary surgery, alternative techniques have been proposed, such as the inverted ILM flap technique in which instead of completely removing the ILM, a remnant attached to the margins of the MH is left in place. This ILM remnant is then inverted upside down to cover the MH [55]. With the use of this technique closure rates of 98% compared to 88% with the standard technique have been achieved [55]. For refractory MH to the standard technique or for secondary MH after vitrectomy when peeling of the ILM has been already performed, an autologous transplantation of the ILM remnants introduced into the hole with subsequent gas tamponade contributes to the improvement of anatomic and visual outcomes [56].

#### 3.3.2. Vital Dyes for ILM Staining

Vital dyes have become effective and useful tools for identifying ocular tissues during vitrectomy, thereby facilitating ILM peeling and ensuring complete removal of this delicate membrane [57]. The most frequently used vital dyes include triamcinolone acetonide suspension in balanced salt solution (BSS) (Triesence), indocyanine green and infracyanine green, brilliant blue, and trypan blue with brilliant blue (Membrane Blue-Dual).

Triamcinolone suspension in BSS is not a true dye but is very useful for the identification of vitreous remnants and the posterior hyaloid. Deposition of crystals on the ILM surface helps the achievement of complete removal of the membrane, although it is less effective than vital dyes because triamcinolone does not increase the rigidity of ILM.

Indocyanine green and infracyanine green possess a great affinity for the matrix components of the ILM and produce intense staining of the ILM. Besides the ability of indocyanine and infracyanine green to stain the ILM, they cause an increase in the biomechanical stiffness of the ILM, thereby facilitating its peeling. Although in Europe they are no longer used because of potential toxicity, they continue to be used in the United States [58, 59].

Brilliant blue has a remarkable affinity for the ILM and, although ILM staining is less intense than that achieved with indocyanine green, causes adequate staining of the ILM and may be used without fluid-air exchange. In Europe, it is considered the best one for ILM peeling in MH surgery.

The combination of trypan blue and brilliant blue allows staining of the ERM, posterior hyaloid, and ILM simultaneously. This combination has a lower density than water and BSS and circumvents the need for fluid-air exchange. This dual dye is extensively used in Europe [60].

#### 3.3.3. Tamponade and Postoperative Positioning

There is controversy regarding posturing in MH surgery. Although most authors recommend face-down posturing 90% of time for 10 days, different studies have reported successful hole closure in the absence of face-down positioning, given that isolation of the macula by gas tamponade maintaining the macula dried seems to be the most important factor for closure [61–63]. In this respect, OCT studies have shown that hole closure occurs during the first postoperative day independently of the types of gas tamponade and posturing [64], so that after vitrectomy with wide ILM peeling, gas tamponade would be sufficient (preferably short-acting gases, such as SF6) at nonexpansible concentration, without the need of face-down posturing, avoiding the prone position during 3 to 5 days. This approach may be also indicated for phakic patients because it does not seem to increase the incidence of cataracts [54].

#### 3.3.4. Combined Phacovitrectomy

Combined phacovitrectomy or sequential vitrectomy and phacoemulsification are safe and effective for the treatment of MH, with equivalent anatomic and functional results [65]. In most cases, idiopathic MH affects patients older than 50 years in which some degree of lens opacity is frequent. Moreover, cataract develops in 75% to 95% of patients undergoing vitrectomy for MH within 3 years after surgery. For this reason, most authors recommend combined phacovitrectomy in patients over 50 years of age. Both cost and discomfort are lower with a single surgical procedure, and functional recovery is more rapid. Combined phacovitrectomy may also decrease the risk of reopening after cataract extraction in the two-step surgical approach [66, 67]. However, combined vitrectomy, phacoemulsification, and intraocular lens (IOL) implantation may be associated with complications, including a high degree of postoperative anterior chamber inflammation and a higher risk of IOL dislocation or papillary capture, generally as a result of excess gas tamponade and/or poor compliance to positioning [68]. Therefore, the decision of the combined versus the two-step procedure should be individualized according to the characteristics of each case and the patient's and surgeon's preferences.

### 3.4. Results of Surgery for MH and Complications

In the study of the* Moorfields Macular Hole* (MMHS) Group [69], an overall closure rate of 81% at 2 years was achieved in MHs stages 2, 3, and 4 as well as an improvement in visual acuity of 6/36 to 6/18, which was clearly superior to results obtained in the observation group. In the* Vitrectomy for Treatment of Macular Hole Study* (VMHS), the rate of anatomic closure was 69% and the final visual acuity was higher in the operated than in nonoperated eyes (20/115 versus 20/166) [70].

Once peeling of the ILM has become popular, closure rates of 90% to 100% were reported [71–75]. However, the use of indocyanine green was associated with potential toxicity in some cases [76] and, for this reason, trypan blue and brilliant blue are in widespread use in some countries, with closure rates of 94% to 100%, without apparent severe side effects [77–80]. Despite its clear indication and safety in MH surgery, ILM peeling is a traumatic procedure that has acute effects on the underlying retinal nerve fiber layer. ILM peeling often results in temporary swelling of the arcuate nerve fiber layer (SANFL) which may be the earliest manifestation of dissociated nerve fiber layer (DONFL) which occurs later in the postoperative period. However it is probably a transient feature that does not affect visual recovery [80].

Although peeling of the ILM has been widely adopted in MH surgery, the high percentages of hole closure obtained in the years prior to systematic ILM peeling add uncertainty about whether to use it in all cases. Recently, Spiteri Cornish et al. [81, 82] carried out a systematic review and meta-analysis to assess the success of HM surgery with ILM peeling compared with the nonpeeling technique. Four randomized clinical trials comparing both techniques were identified [48, 49, 51, 81, 82]. There was no evidence of a difference in the primary outcome (distance visual acuity at six months), nor in distance visual acuity at 12 months between randomized groups. Overall, 66.2% achieved a visual acuity equal or greater than 69 letters on ETDRS charts (corresponding Snellen visual acuity 20/40) and 77.9% gained more than three ETDRS lines. Improvement of visual acuity was higher in patients in which primary anatomic closure was achieved (final visual acuity 72.8 ± 7.6 letters and a mean improvement of 21.6 ± 7.1 letters) than in eyes in which further surgery was required (66.4 ± 8.6 and 17.4 ± 7.7 letters, resp.) [49].

However, visual improvement was obtained somewhat earlier in the ILM peeling group and, at 3 months, improvement was greater if ILM peeling was performed. In addition, the percentage of primary closure was higher in the ILM peeling as compared with no peeling (89.9% versus 50.3%, with an odds ratio (OR) of 9.27 and 95% confidence interval [CI] of 4.98–17.24). When reoperations were excluded from the analysis, the ILM peeling group continued to have more favourable results (OR 3.99, 95% CI 1.63–9.75). Also, in MH stage 2, the efficacy rate was better for ILM peeling than no peeling (91.6% versus 61.3%, with an OR of 6.19; 95% CI 1.65–23.20) [48, 49, 83].

This higher success rate was not accompanied by an increase of perioperative complications, neither in the reports in which the ILM was stained with indocyanine green. In the meta-analysis [81], the rate of intraoperative complications was 19.32% for the ILM peeling group as compared with 21.1% for the nonpeeling group (OR 0.94, 95% CI 0.47–1.87). The most frequent intraoperative complications were small retinal hemorrhage (6–19%), retinal tears (5.4–32%), retinal detachment (2–6%), and choroidal hemorrhage (0–3%).

According to these data, the authors conclude that ILM peeling offers more favourable cost-effectiveness compared with no peeling in MH surgery [81, 82].

### 3.5. Surgery-Related Prognostic Factors and Management of Reopening

Although anatomic closure in MH surgery is achieved in more than 90% of cases, sometimes it does not correlate well with improvement in visual acuity. Multiple studies using OCT have assessed hole configuration in an attempt to establish a correlation with postoperative visual acuity [84–91], emphasizing the importance of changes in the outer retina. Kusuhara et al. [84] defined a macular hole index (MHI) as a ratio of hole height to base diameter of hole, calculated from OCT transverse images of the macular area, establishing that a MHI ≥0.5 was correlated with better postoperative visual acuity than MIH <0.5. Ruiz-Moreno et al. [85] described the diameter hole index (DHI) as a ratio between minimum hole diameter and base diameter, showing the minimum diameter was the best preoperative predictive prognostic factor.

Different studies have shown a direct correlation between integrity of the hyperreflective line as IS/OS junction of photoreceptors and postoperative improvement of visual acuity. In the study of Kitaya et al. [86], postoperative vision ≥0.7 was correlated with good reconstitution of the IS/OS junction. However, Sano et al. [87] showed that a continuous IS/OS line was not a reliable prognostic factor in the early postoperative period given that abnormalities of the IS/OS line seen on SD-OCT can be gradually repaired, with achievement of a continuous IS/OS line at 6 months. Spaide and Curcio [88] assessed the correlation of the outer retina analyzed by means of SD-OCT and histopathological findings, showing that the hyperreflective line identified as IS/OS junction of photoreceptors corresponded to the ellipsoid portion of the photoreceptor inner segment, containing mitochondria. Wakabayashi et al. [89] using SD-OCT described that reconstitution of the external limiting membrane (ELM) was more important to predict subsequent restoration of the foveal photoreceptor layer than the ellipsoid zone restoration. Restoration of ELM seems to be a necessary factor for reconstitution of the ellipsoid band, with subsequent migration of photoreceptors and complete closure of the full-thickness MH. Ruiz-Moreno et al. [90] have analyzed 164 eyes with MH treated by vitrectomy and ILM peeling showing that restoration of the ellipsoid portion of the photoreceptor inner segment is an important prognostic factor for visual rehabilitation after MH surgery.

**Figure 4:**
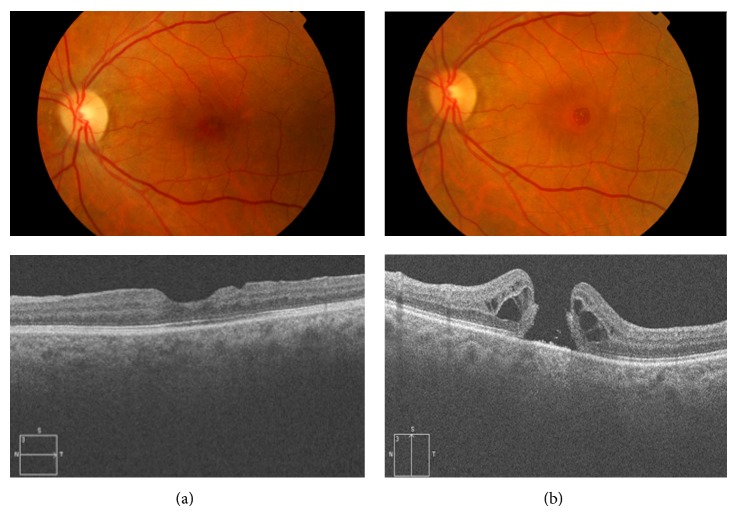
(a) Fundus photography and OCT of a patient who underwent macular hole surgery with ILM peeling and adequate reconstitution of the outer retina (ELM and ellipsoid bands) and visual acuity 20/30. (b) Reopening of the MH after 3 years with cystoid edema surrounding the hole and decreased visual acuity to 20/200. The ERM is not observed.

Reopening of the hole (Figure 4) is one of the best known complications after initially successful MH treatment with vitreous surgery [67, 91–101]. Peeling of the ILM during primary MH surgery is one of the factors that has been mostly related to the incidence of reopening, varying between 0% and 8% in eyes with ILM peeling [67, 95–97] and between 2% and 16% in eyes with no peeling [67, 93–95, 97]. The variable percentages reported in the studies are due in part to differences in the length of follow-up, with higher rates associated with prolonged follow-up periods. Paques et al. [93] reported a 9.5% incidence with a mean follow-up of 2 years, whereas Scott et al. [94] found a 12% incidence with a mean follow-up of 7 years. Kumagai et al. [95] analyzed the results of surgery in a series of 877 cases of MH, increasing the reopening percentage to 28.1% with no ILM peeling. The incidence of recurrence was 0.39% in eyes with peeling of the ILM, increasing to 7.2% with no peeling. Besides no peeling, statistically significant risk factors for reopening were myopia of more than 6 diopters and intraoperative retinal tears. Retinal tears treated with laser may be one of the factors that increase the development of ERM, with subsequent tangential traction and reopening of the MH.

No peeling of the ILM may be associated with a higher risk of ERM formation [92, 93, 97, 100]. Yoshida and Kishi [97] observed the presence of ERM in all cases of reopening of the hole. However, Kumagai et al. [95] did not report ERM in none of the cases with hole reopening assessed by SD-OCT.

In relation to the incidence of reopening with bilateral MH, Duker et al. [92] reported bilateral reopening in 38% of cases, Christmas et al. [99] in 59%, Scott et al. [94] in 38%, and Kumagai et al. in 14.9% [95].

Cataract surgery in the postoperative period of MH surgery has been involved in the reopening of MH. Paques et al. [93] observed that 73% of cases of hole reopening occurred after a secondary cataract surgery. Bhatnagar et al. [67] reported that in the presence of cystic macular edema after secondary cataract surgery, there was a sevenfold increase in the risk of reopened holes, and García-Arumí et al. [101] reported recurrence of MH reopening after posterior capsulotomy. However, other authors, including Kumagai et al. [95] and Sheidow and Gonder [102] reported cystoid edema in combined surgical procedures and that the incidence of hole reopening did not increase in secondary cataracts.

**Figure 5:**
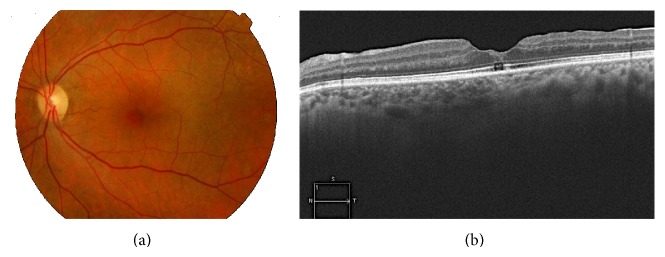
Fundus photography and OCT after reoperation using platelet-derived growth factors. Successful anatomic hole closure is observed but glial type scar in the inner retina and the absence of a continuous ellipsoid band determined a final visual acuity of 20/60.

With regard to treatment of persisting MH, ILM peeling and ERM removal should be performed in those cases in which these procedures were not performed at the initial macular surgery, together with long-acting gas tamponade (C3F8) and strict face-down positioning during the first postoperative days. In these patients, the anatomic and functional success is high. When ILM peeling and removal of the ERM have been performed in the first surgical procedure, the success of reoperation decreases. In a series of 30 patients reported by D'Souza et al. [103] with initial ILM peel who underwent repeat surgery involving vitrectomy, enlargement of ILM rhexis, and gas tamponade with C3F8, the anatomic closure rate was 88% for primary surgery and 46.7% for reoperation. More extensive ILM peeling causing tangential traction due to fibrosis of dissection margin may contribute to the anatomic closure. The use of growth factors, such as platelet-derived growth factors as a stimulus of glial progenitor cells, may be useful if the ILM has been adequately peeled (Figure 5), as well as the use of heavy silicone oil in patients with positioning difficulties [104].

## 4. Practical Considerations: Therapeutic Algorithm

**Figure 6:**
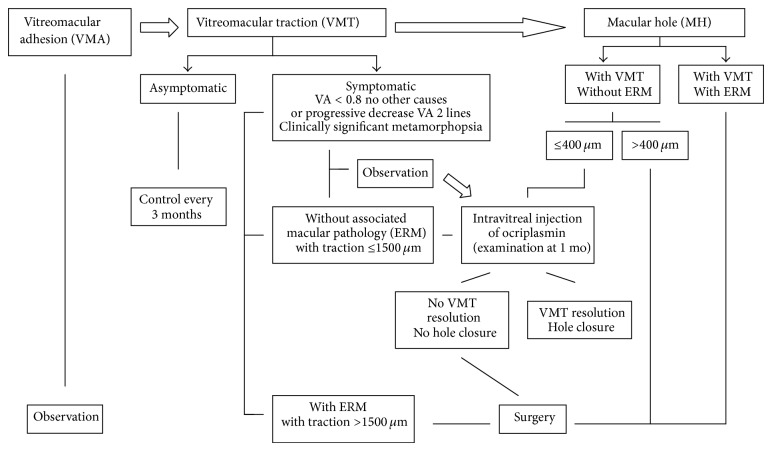
Treatment algorithm for VMA, VMT, and MH (VA: visual acuity, ERM: epiretinal membrane).

Based on the aforementioned data and as shown in the schematic representation in Figure 6, patients with VMA can be observed without the need of any intervention. In cases of VMT it is crucial to take into account the patient's symptoms. If the patient is asymptomatic, a follow-up control at 3 months may be sufficient. During this interval, the patient should be advised to perform periodic self-examinations with the Amsler grid or monocular reading tests. In case of symptoms, intensity and disability should be assessed. There is no consensus criterion regarding the degree of vision loss that should be considered significant and amenable to treatment. However, in the TG-MV-006 y TG-MV-007 clinical trials, patients with visual acuity equal or lower than 20/25 were eligible [20], so that this level of visual impairment can be already considered to be susceptible of treatment. Also, other causes that may justify decreased visual acuity should be excluded. Metamorphopsia clinically significant for the patient and visual loss progression are also key factors at the time of adopting a more interventional therapeutic attitude. Despite these considerations, a period of observation may be an option for these patients, because spontaneous resolution is still possible. In case of deciding an active treatment, the presence of other associated macular diseases, such as ERM, should be excluded [20]. When traction is ≤1500 *μ*m, enzymatic vitreolysis with ocriplasmin is the treatment of choice. In the presence of >1500 *μ*m traction or ERM, surgical treatment with vitrectomy is associated with better outcomes [105].

In cases with a full-thickness MH, it is necessary to assess the diameter size. In cases of holes ≤400 *μ*m in size with MVT and in the absence of ERM, enzymatic vitreolysis with ocriplasmin is again the most recommendable option [20]. In cases of holes >400 *μ*m, or in the absence of evident VMT, or in the presence of ERM, vitrectomy is the first option [105].

Patients undergoing enzymatic vitreolysis with intravitreal injection of ocriplasmin should be evaluated at 7 and 30 days. Most cases of VMT or MH resolve within the first week of treatment [20] and also at this time the occurrence of potential treatment-related complications should be excluded. If resolution of VMT and/or hole closure had not occurred after a month of treatment, the likelihood of success is highly improbable and vitrectomy can be performed. Patients with lamellar MH or pseudomacular holes in which traction is usually absent are also candidates for enzymatic vitreolysis. In cases of VMT associated with other retinal diseases, such as age-related macular degeneration, diabetic macular edema, or vitreomacular interface pathology in the myope, it is still too early to make a recommendation on the impact of enzymatic vitreolysis with ocriplasmin in the treatment of these conditions, and we should await for results of ongoing clinical trials on this topic [106].

Finally, in all cases, the final decision regarding treatment with enzymatic vitreolysis with ocriplasmin or vitrectomy should be consensuated with the patient. All cases in which the use of ocriplasmin is considered a first treatment option can be successfully treated by means of vitrectomy. Also, it may be possible that patients who initially are not ideal candidates for enzymatic vitreolysis may have their pathologic condition solved by treatment with ocriplasmin [20]. Favourable prognostic factors for the choice of vitreolysis have been identified including young age and phakic status, but difficulties to maintain postoperative face-down posture or the waiting lists for vitrectomy are variables that should also be considered.

## 5. Conclusions

Enzymatic vitreolysis based on the intravitreal injection of ocriplasmin is a treatment option with proven efficacy and adequate safety profile in selected patients with VMT and MH. In cases of VMT, treatment with ocriplasmin is indicated when traction is ≤1500 *μ*m and in the absence of concurrent macular diseases (ERM). In the case of MH, the hole diameter should be ≤400, traction has to be present, and ERM should be absent. When resolution of the process after one month of the procedure is not achieved, vitrectomy with ILM peeling would be the surgical treatment of choice.

## Figures and Tables

**Table 1:** 

Gass classification	OTC findings	Classification IVTS
Stage 0	Minimal changes in the foveal contour with perifoveal detachment of the perifoveal vitreous cortex without traction	VMA

Stage 1A: imminent MH	Foveal cysts and sensory foveolar detachment associated with perifoveal detachment with traction of the posterior vitreous on the foveal internal limiting membrane	VMT

Stage IB	Cyst in the outer retina causing rupture of the cones layer. Perifoveal detachment of posterior vitreous	VMT

Stage 2: small MH	Full-thickness MH of small diameter, with partial rupture of the internal wall of the cyst. Partial detachment of the posterior vitreous, which still remains adhered to the operculum	FTMH small/medium with VMT

Stage 3: large MH	MH of a larger size. Total detachment of the posterior vitreous at the level of the macular area, which persists adhered to the papilla. Occasionally, a free operculum adhered to the posterior vitreous can be seen	FTMH medium/large with VMT

Stage 4: full-thickness MH with PVD	Total detachment of the posterior vitreous. In some cases, the vitreous is not observed on OCT scans. Larger diameter of the hole with halo of outer retinal detachment in many occasions	FTMH small/medium/large without TVM

FTMH: full-thickness macular hole, MH: macular hole, OCT: optical coherence tomography, PVD: posterior vitreous detachment, VMA: vitreomacular adhesion, and VMT: vitreomacular traction.
